# Rethinking the potential and necessity of drug delivery systems in neovascular age-related macular degeneration therapy

**DOI:** 10.3389/fbioe.2023.1199922

**Published:** 2023-05-23

**Authors:** Xi Huang, Li Zhang, Yanyan Fu, Meixia Zhang, Qian Yang, Jinrong Peng

**Affiliations:** ^1^ Department of Ophthalmology, Research Laboratory of Macular Disease, Department of Biotherapy, Cancer Center and State Key Laboratory of Biotherapy, West China Hospital, Sichuan University, Chengdu, Sichuan, China; ^2^ Center of Scientific Research, Chengdu Medical College, Chengdu, Sichuan, China

**Keywords:** age-related macular degeneration (AMD), drug delivery system, long-term delivery, pathogenesis, intravitreal delivery

## Abstract

Age-related macular degeneration (AMD) is the predominant threat to human vision and ultimately results in blindness. With the increase in the aging population, it has become a more crucial issue to human health. AMD is a multifactorial disease with the unique feature of uncontrollable angiogenesis during initiation and progression. Although increasing evidence indicates that AMD is largely hereditary, the predominant efficient treatment is antiangiogenesis, which mainly involves VEGF and HIF-α as therapeutic targets. The repeated administration of this treatment over the long term, generally through intravitreal injection, has called for the introduction of long-term drug delivery systems, which are expected to be achieved by biomaterials. However, the clinical results of the port delivery system indicate that the optimization of medical devices toward prolonging the activities of therapeutic biologics in AMD therapy seems more promising. These results indicate that we should rethink the possibility and potential of biomaterials as drug delivery systems in achieving long-term, sustained inhibition of angiogenesis in AMD therapy. In this review, the etiology, categorization, risk factors, pathogenesis, and current clinical treatments of AMD are briefly introduced. Next, the development status of long-term drug delivery systems is discussed, and the drawbacks and shortages of these systems are emphasized. By comprehensively considering the pathological aspect and the recent application of drug delivery systems in AMD therapy, we hope to find a better solution for the further development of long-term therapeutic strategies for AMD.

## 1 Introduction

**FIGURE 1:**
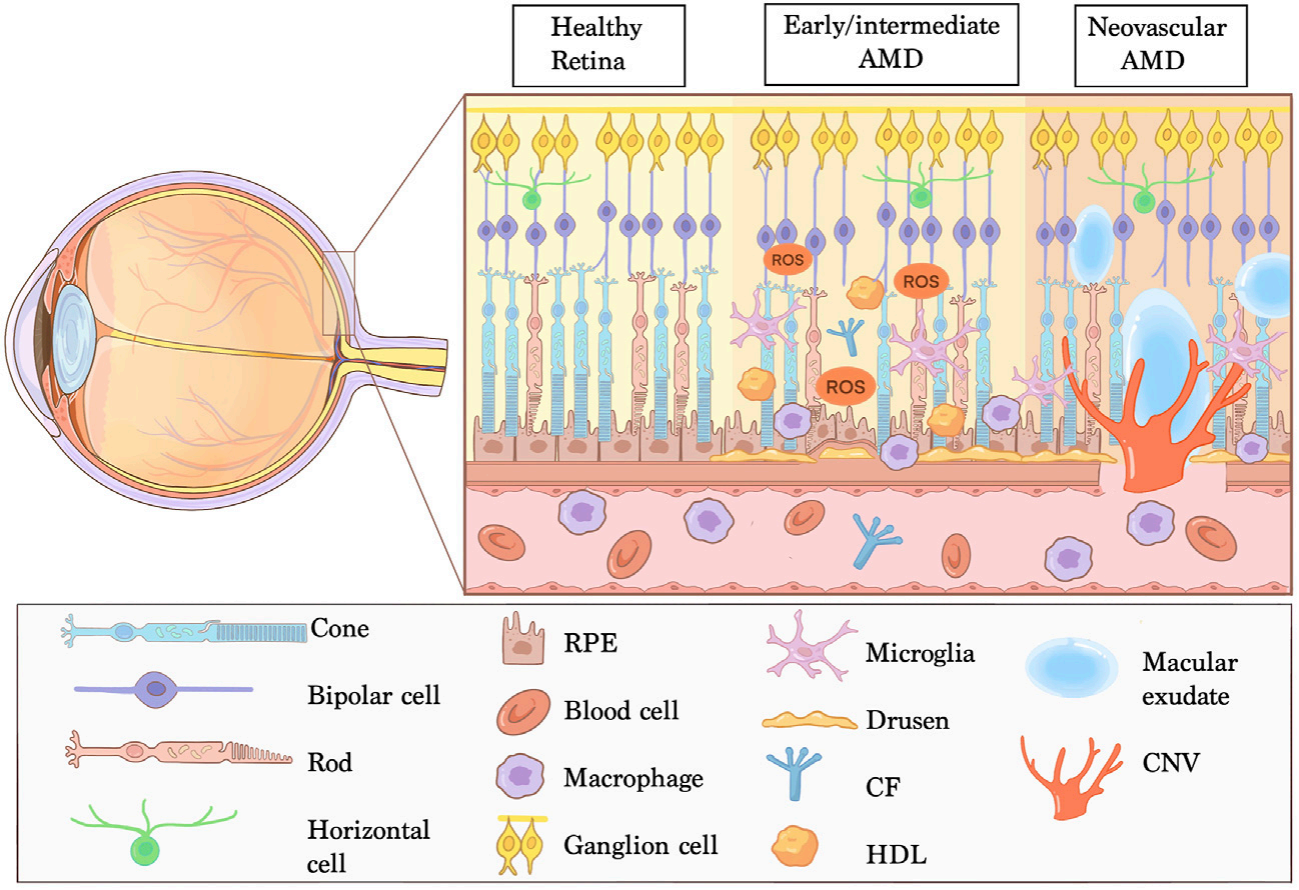
Manifestations of NVAMD. Age-related macular degeneration (AMD) is a multifactorial disease affecting the complex of photoreceptors, retinal pigment epithelium (RPE), Bruch’s membrane and choriocapillaris. The retina-RPE-Bruch’s membrane choroid complex is normal in healthy retina. The early and intermediate AMD are typical of deposition of extracellular debris named Drusen and pathological changes in the RPE. Neovascular AMD is characterized by invasion of choroidal neovascularization (CNV) into the outer retina, subretinal space or sub-RPE space and imbalanced angiogenesis factors. In addition, chronic inflammation, lipid deposition, oxidative stress and impaired extracellular matrix maintenance are strongly associated with AMD pathogenesis. Abbreviations: CF, complement factors; HDL, high density lipoproteins; ROS, reactive oxygen species.

Age-related macular degeneration (AMD) remains the leading cause of low vision and blindness in among geriatrics over 55 years worldwide (Wong et al., 2014; Mitchell et al., 2018). In early- and intermediate-stage AMD, the pathological event is deposits of lipoprotein called drusen, or diffused form as basal linear deposits (BLinD), which commonly locates in subretinal space, along with overlying photoreceptors and retinal pigment epithelium (RPE) degeneration. AMD progresses to advanced form, designated as geographic atrophy (GA) or neovascular AMD (NVAMD) (Apte, 2021). NVAMD is the main clinical type, occupying 10%–20% of AMD and inducing severe loss of center vision while pathologic new blood vessel growth into the macula. This “leaking” vessel development is called choroidal neovascularization (CNV), which results in repeated macular bleeding, exudation, and fibrosis, potentially permanently damaging the morphology and function of the macula (Figure 1).

Since 2016, anti-vascular endothelial growth factor (VEGF) therapy have remarkably ameliorate vision prognosis and life quality of NVAMD patients (Campochiaro et al., 2016). However, a significant proportion of patients with NVAMD present with persistent exudation, unresolved or new hemorrhages, and progressive fibrosis, and vision cannot be restored (Mettu et al., 2021). Additionally, the predominate phenomenon of choriocapillaris dropout found by newly developed retinal imaging modules like optical coherence tomography (OCT) and OCT-Angiography strongly sustain histopathologic discoveries in NVAMD patients. This may indicate that hypoxia and VEGF upregulation are less impossible self-eliminate (McLeod et al., 2009; Mullins et al., 2011; Biesemeier et al., 2014). Exudation, one aspect of disease activity, could be worsened while the agent is cleared. Additionally, repeated intravitreal injections in a long term can lead to complications, such as elevated intraocular pressure, uveitis, retinal detachment and endophthalmitis (Mehta et al., 2018). Thus, there is an urgent need to develop alternatives, which have low adherence, high socioeconomic costs, and long-lasting or sustained release of anti-angiogenesis drugs. In this review, we will briefly discuss the clinical aspects and treatment modalities of NVAMD and highlight the progress and future perspectives of nanotherapeutics.

## 2 Epidemiology, categorization, and risk factors for NVAMD

### 2.1 Epidemiology of NVAMD

A systematic review and meta-analysis including 12, 727 AMD cases from 39 studies based on a population of 129, 664 individuals showed a prevalence of 8.69%, 8.01% and 0.37% related to any, early and late AMD respectively, the estimated number of patients with AMD in 2020 is 196 million, increasing to 288 million in 2040 (Wong et al., 2014). Furthermore, population increase by 38% globally between 2000 and 2050, among those the proportion of individuals older than 60 will increase by 400%; the number of individuals with NVAMD is estimated to be 80.4 million by 2050, posing a major public health problem with substantial socioeconomic implications (Smith, 2010).

### 2.2 Categorization of NVAMD

In 2020, an international study group discussed and codified a set nomenclature framework for classifying the subtypes of neovascular AMD and associated lesion components (Spaide et al., 2020). The consensus is that neovascularization does not necessarily originate from the choroid in patients with NVAMD. Compared to CNV, macular neovascularization (MNV) is a more specific term describing the pathologic blood vessel of NVAMD. The group proposed three categories of MNV (Spaide et al., 2020): Type 1 and Type 2 MNV refer to neovascularization that originates from the choroid, the former grows under the sub-RPE space leading to different types of pigment epithelium detachment (PED), while the latter broke through RPE-Bruch’s membrane (BrM) into the subretinal space. Type 3 MNV refers to neovascularization that originates from the deep capillary plexus of retinal circulation and grows toward the outer retina. The same treatment may have different efficacies in different subtypes of NVAMD, indicating the complexity of its pathogenesis, which deserves more exploration in the future.

### 2.3 Risk factors for NVAMD

Many risk factors have been associated with AMD (including both non-neovascular AMD and NVAMD), with aging being the strongest, making AMD a multifactorial, spectrum of disease. In addition to age, AMD is strongly susceptible to genetic variants. Genetic factors account for 40%–60% of all AMD attribution risk, and variants in CFH and ARMS2–HTRA1 confer the highest risk of AMD (Seddon, 2017; Mitchell et al., 2018). AMD risk is also influenced by nongenetic and environmental factors, such as smoking and diet (Lambert et al., 2016; Seddon, 2017). Smoking is the most important modifiable risk factor for AMD, and the pathogenesis of smoking as a risk factor may include choroidal thinning, oxidative stress, and activation of compliment factors (Myers et al., 2014). Increased overall fat intake accelerates the development of AMD, while increasing ω-3 fatty acid intake in the diet, such as fish, reduces the risk of AMD (Tan et al., 2008; Zhu et al., 2016). Cardiovascular events like hypertension and hyperlipidemia have been inconsistently related to AMD (Cheung and Wong, 2014). Serum lipids concentration increased risk of intermediate AMD in some studies but not in others (Kabasawa et al., 2011; Pennington and DeAngelis, 2016).

## 3 Pathogenesis of NVAMD

In recent years, due to the in-depth study of AMD, we have a deeper understanding of its pathogenesis, and the pieces of puzzles have gradually formed a clear network of etiology. Age and lipid metabolism, genetic susceptibility, inflammation, innate immunity, and angiogenesis, these pathological events promote and interact with each other, leading to the terminal stage of AMD disease, namely, NVAMD. Moreover, there is growing evidence that inflammatory activation plays an important role in the pathogenesis of NVAMD.

### 3.1 Ageing related changes to RPE-BrM-choriocapillaris complex

RPE-BrM-choriocapillaris complex and photoreceptor are the key structures in pathogenesis of AMD. They are anatomic adjacent and metabolic dependent to each other. REP dysfunction is considered as a trigger for early AMD, but not a single event. In normal aged RPE cells, a series of physiological changes include: multinucleation, decreased mitochondrial numbers, accumulation of lipofuscin, and et cl (Tong et al., 2022). These changes make RPE cells more sensitive to oxidative stress and cytotoxicity such as smoking and other toxic substances. Lipid metabolism abnormality mainly refers to the accumulation of lipoprotein in the cell. N-retinylidene-N-retinylethanolamine (A2E) is a well-known fluorophore of lipofuscin contributing to RPE cells degeneration (Sparrow and Boulton, 2005). Photosensitization of A2E can increase DNA damage and cells senescence which could affect the microenvironment of the retina by expressing several factors of the secretory phenotype including IL1B, IL13RA2, and CXCR4 through the NF-κB pathway, expression of these factors also may create a pro-inflammatory environment that drives retina degeneration (Wang et al., 2018). Meanwhile, Bruch’s membrane is responsible for fluid exchange between out layer retina including RPE and choriocapillaris. With ageing, Bruch’s membrane becomes thicker and more fragile, which may weaken capacity of transportation resulting in lipid accumulation. The rupture of Bruch’s membrane is considered to a must condition during the formation of choroidal neovascularization. Age-related changes of choriocapillaris mainly manifest the decreased of vessel density and widened distance between the lumens (Ramrattan et al., 1994; Spraul and Grossniklaus, 1997). This phenomenon of choriocapillaris drop out make clearance of lipid particles and cell-debris less sufficient, further generate hypoxia in the complex of RPE-BrM-choriocapillaris, an important prerequisite for angiogenesis.

### 3.2 Complement activation

Variants of genes encoding complement system regulatory proteins (e.g., CFH, CFI, C3 and FB/C2) contain major risk susceptibility alleles for AMD (Seddon, 2017; Toomey et al., 2018). The complement system is part of the innate immune system whose main role is to recognize and identify foreign pathogens, apoptotic cells, and cellular debris. Activation of the complement system revolves around the cleavage of C3. C3 produces C3a and C3b under the action of invertase (Kato et al., 2020). Alternative pathways also exist with automatic activation, which is controlled by C3 invertase degradation. The alternative pathway is automatically activated when C3 invertase degradation is weakened, and one of the main cofactors for C3 invertase degradation is complement factor H (CFH). When the alternative pathway is activated, large amounts of C3b are produced, which in turn induces the formation of C5 convertase and membrane attack complex (MAC). MAC stimulates signaling pathways related to tissue remodeling, inflammation, and vascularization (Park et al., 2021).

C3 and other complement proteins can be detected in the composition of drusen, indicating that drusen itself will further stimulate inflammatory response and C-reactive protein, and local inflammation caused by abnormal RPE will aggravate drusen aggregation (Bhutto et al., 2011). There is also a lot of evidence that complement activation first occurs in the choroidal capillary layer. Complement activation also stimulates the natural inflammatory response of choroidal endothelial cell. Choroidal capillaries are the only capillaries that express the intercellular adhesion factor ICAM-1 in endothelial cells, which mainly regulates leukocyte infiltration. *In vitro* studies have shown that C5a can increase the expression of ICAM-1, indicating that complement activation can promote choroidal capillary leukocyte recruitment (Johnson et al., 2006; Skeie et al., 2010).

### 3.3 Inflammation

Low-level inflammation (parainflammation) is essential for maintaining homeostasis and monitoring dysfunction of cells and tissue. Once the optimal homeostatic balance is broke, excessive parainflammatory responses may lead to chronic inflammation and subsequent tissue dysfunction (Copland et al., 2018). AMD patients show high expression of inflammatory/immune-related factors in the eyes, retina and choroid can recruit many macrophages and microglia (Wooff et al., 2019). These are distributed along nonimmune cells (RPE and Müller cells) and participate in disease development (Nussenblatt et al., 2013). Macrophages have different effects, with macrophages in the young choroid helping Bruch’s membrane to clear precipitation and cholesterol, whereas macrophages may also have a destructive effect with age, participating in the formation of CNV by stimulating high expression of VEGF-A (Cherepanoff et al., 2010). According to the functional phenotype of polarization, macrophages can be divided into the classical activating type with anti-angiogenesis function (M1) and the surrogate-activated type with pro-angiogenesis function (M2) (Funes et al., 2018), and the M1/M2 macrophage ratio can regulate the development of CNV (Lutty and McLeod, 2018). Transition of macrophages from a protective mechanism to a destructive mechanism may be related to the disruption of the build-clear homeostasis of lipid oxidation products with age. In addition, studies in humans and rodents have shown that macrophages are the main infiltrating cells of CNV (Campochiaro, 2015). IL-6 produced by macrophages stimulates CNV formation by classical activation of IL-6 receptor (IL-6R)-positive macrophages (Droho et al., 2021). Another immune cell was found to exist each stage of AMD is mast cell, and some researchers have speculated that mast cells may be involved in regulating pro-inflammatory and anti-inflammatory responses, resulting in damage to the vascular wall and Bruch’s membrane, RPE degradation and thinning of the choriocapillaris and other pathological changes that promote CNV production (Bhutto et al., 2016).

### 3.4 Angiogenesis

**FIGURE 2:**
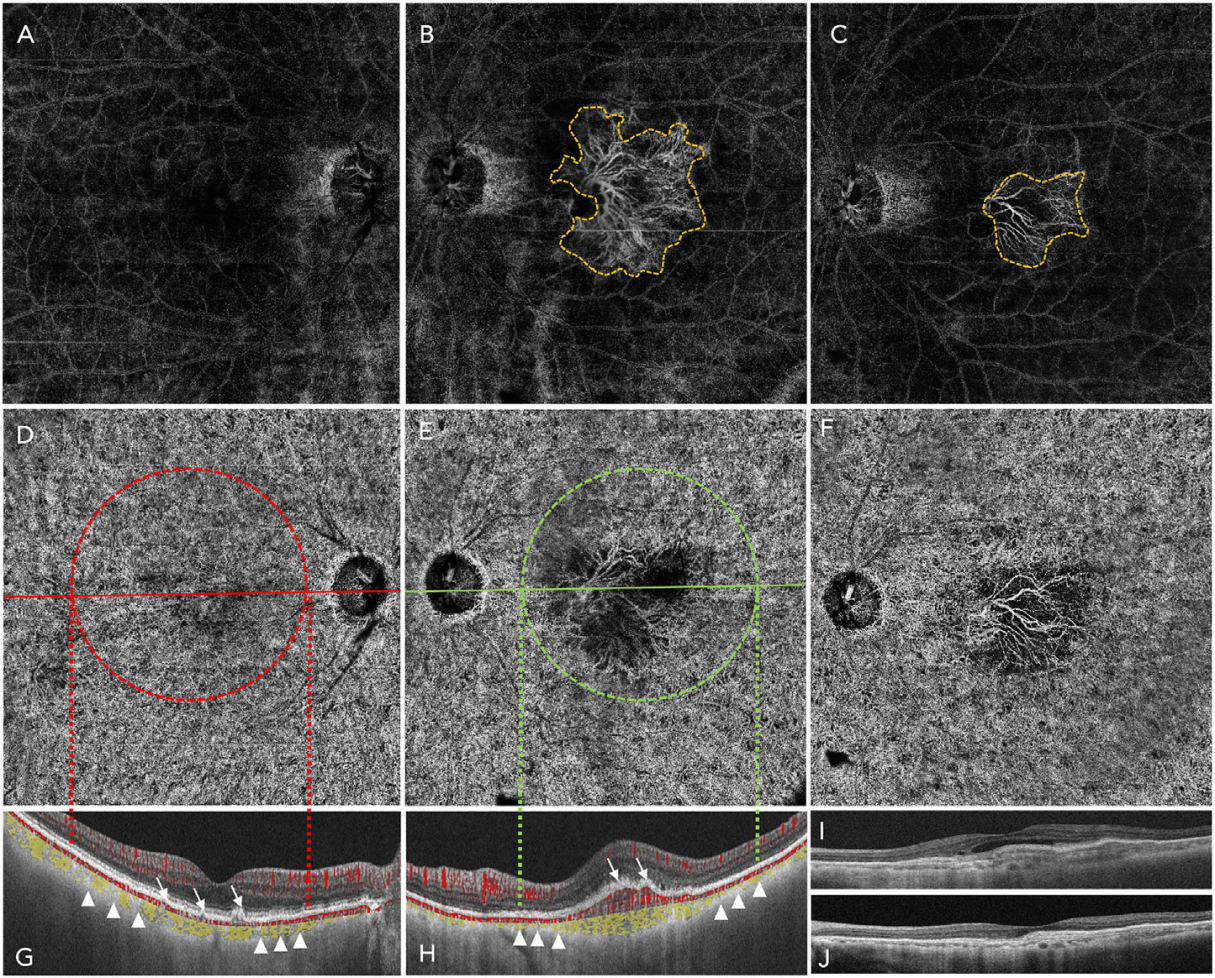
A 12 mm × 12 mm wide-field swept-source optic coherence tomography angiography (SS-OCTA) image of an 80-year-old woman who complained of vision loss of her left eye for 2 weeks. She was diagnosed of early AMD of right eye and NVAMD of left eye. **(A–C)** shows the avascular lay (defined by the software from the out plexiform layer to Bruch’s membrane of the retina): no abnormal blood vessel grows in the right eye **(A)**; the yellow dashed line outlines a fan-like treat-naïve type 2 choroidal neovascularization (CNV) of the left eye **(B)**; 1 month after the patient received an intravitreal anti-VEGF therapy of Aflibercept 2.0 mg, the area of type 2 CNV reduced markedly compared to the baseline **(C)**. **(D,E)** shows the choriocapillaris layer. **(G,H)** shows the B-scan of the right eye **(G)** and left eye **(H)** accordingly: the white arrows of the right eye indicates the deposition of extracellular debris (drusen within which there is no blood signals) and the white arrows of the left eye indicates the CNV invasion into the subretinal space with blood signals (red), and the yellow area lining with the choroid shows that the NVAMD eye is thinner than the early stage AMD eye (small white triangles), this is the phenomenon of the drop out of choriocapillaris of NVAMD. **(I,J)** showed the B -scan of the left eye: after treatment, the intraretinal edema was absorbed, and the central retina thickness reduced.

Recent clinical and histologic features studies suggests that non-exudative Type 1 CNV acts as a compensation mechanism, providing nutritional support and functional maintenance for outer retinal photoreceptors. This mechanism mainly causes by aggravating hypoxia in the complex of RPE-BrM-choriocapillaris, induced by the drop out of choroidal capillaries, and accumulation of local lipid components and cellular fragments which hinder exchange of oxygen and nutrients. When the compensatory equilibrium is broken, neovascularization occurs, breaking through BrM to form type 2 CNV (Chen et al., 2020) (Figure 2).

The pathogenesis of CNV is complex, and vascular endothelial growth factor (VEGF) is one of the main regulators of angiogenesis in NVAMD, specifically acting on vascular endothelial cells to promote proliferation, induce the generation of new blood vessels, and increase vascular leakage (Campochiaro, 2015). VEGF is mainly bound to vascular endothelial growth factor receptor 1 and 2 (VEGFR-1 and VEGFR-2), which is widely distributed in chorioretinal endothelial cells (Simons et al., 2016). During the cascade of angiogenesis, binding to VEGFR-2 is the most important process (Ferrara et al., 2003). VEGFR2 in endothelial cells is transported to the cell surface, and its tyrosine kinase can be activated by VEGF-A to induce angiogenesis and increase vascular permeability (Ferrara et al., 2003; Waters et al., 2021). VEGFR1 can be used as bait to weaken VEGF-A-mediated angiogenesis and leakage. In addition, VEGFR1 activation leads to neovascularization, increases vascular permeability, and induces macrophages and microglia to produce proinflammatory and angiogenic mediators (Uemura et al., 2021).

## 4 Clinical trials of therapies for NVAMD

The first anti-VEGF therapy approved by the FDA in 2004 for the treatment of NVAMD was pegaptanib (Macugen®), a synthesized 28-base oligonucleotide (aptamer) that selectively neutralizes the VEGF_165_ isoform, being modified with two polyethylene glycol (PEG) to increase its intraocular half-life (Gao and Talalay, 2004; Gragoudas et al., 2004). Bevacizumab (Avastin®) is a first-generation humanized monoclonal antibody to VEGF-A that is effective against angiogenesis via systemic and intravitreal injection (Michels et al., 2005). Subsequently, the second-generation monoclonal antibody ranibizumab (Lucentis®) was approved by the FDA for NVAMD by intravitreal administration in 2006 (Rosenfeld et al., 2006). Ranibizumab contains an antigen-binding fragment (Fab) that binds and inhibits all identified human VEGF isoforms (Ferrara et al., 2006). The third FDA-approved drug targeting ocular VEGF is the cytokine trap aflibercept. Aflibercept is a fusion protein composed of the extracellular domain of VEGF1 and VEGF2 receptors bound to the Fc portion of human IgG1 and acts as a decoy receptor, blocking the binding of VEGF to its receptors and inhibiting downstream signaling and angiogenesis (Holash et al., 2002).

The MARINA trial (NCT00056836) enrolled 716 patients. At 12 months, 94.5% of the patients given 0.3 mg of ranibizumab and 94.6% of those given 0.5 mg lost fewer than 15 letters compared with 62.2% of patients receiving sham injections, and the benefit in visual acuity was maintained at 24 months (Rosenfeld et al., 2006; Mitchell et al., 2010). The ANCHOR trial (NCT00061594) reported the inspiring result that ranibizumab was superior to verteporfin as an intravitreal treatment of predominantly classic NVAMD, with low rates of serious ocular adverse events. Treatment improved visual acuity on average at 1 year (Brown et al., 2006).

Although the remarkably good results from all prior trials are exciting, anti-VEGF therapy still faces challenges and limitations. In real-world studies, many patients with NVAMD are not treated at optimal frequency, which is not the same as that observed in precisely designed and conducted clinical trials (Mehta et al., 2018; Rao et al., 2018). In clinical practice, poor compliance also reflects the burden that frequent treatment places on patients and healthcare systems (Cohen et al., 2013; Finger et al., 2013).

To reduce the frequency of IVT or extend the gap of IVT, other strategies are developing. In the VIEW 1 and VIEW 2 trials, intravitreal injection (IVT) of 2 mg aflibercept every 2 months were noninferior to monthly injections of 0.5 mg ranibizumab (Heier et al., 2012). A port delivery system (PDS), a fillable, continuously administered intraocular implant filled with 100 mg/mL ranibizumab for sustained drug release, was developed. However, this PDS device must be surgically sewn into the scleral wall, and the overall price is not advantageous compared to current intraocular injections (Campochiaro et al., 2019). Brolucizumab is a 26 kDa humanized antibody fragment targeting VEGF-A. It has a smaller molecular weight than ranibizumab (48 kDa) and aflibercept (115 kDa), but the molar concentration is 13 times that of aflibercept (Holz et al., 2016). In the HAWK and HARRIER clinical trials, brolucizumab achieved the primary efficacy endpoint of nondisadvantage compared with aflibercept; the central retinal thickness (CRT) was more reduced, the anatomy was better visualized, and 5%–7% of HAWK patients and 5% to 2% of HARRIER patients could maintain a 12-week administration interval up to 48 weeks (Dugel et al., 2020). Among the treated patients who completed 12-week dosing intervals, 82% of patients in the HAWK trial and 75% of patients in the HARRIER trial were in the first 2A stage and still maintained the 12-week dosing interval (Dugel et al., 2020). Brolucizumab has been shown to have a longer persistence in vision and greater anatomical improvement, which is expected to reduce the number of injections in patients and reduce the burden of treatment (Nguyen et al., 2020; Dugel et al., 2021).

## 5 Clinical trials of therapies for NVAMD

### 5.1 Novel anti-VEGF drugs/analogs

Abicipar-pegol (Allergan) is a DARPin molecule that contains engineered ankyrin repeat domain(s) targeting VEGF-A with high specificity and affinity and a longer ocular half-life (Rodrigues et al., 2018). The Phase III SEQUOIA and CEDAR trials (NCT02462928, NCT02462928) recruited patients with treatment naïve NVAMD, and abicipar showed comparable best corrected visual acuity (BCVA) and CRT up to 52 weeks with fewer injections than ranibizumab (Ferro Desideri et al., 2020; Kunimoto et al., 2020). However, because of unfavorable inflammation occurrence and risk-benefit ratio, it did not gain approval from FDA in 2020 (Kunimoto et al., 2020).

OPT-032 is a fusion protein of Fc fragments of VEGFR-3 and human IgG1 for binding VEGF-C and VEGF-D (Joukov et al., 1996). The results of the Phase IIb clinical trial (NCT003345082) presented at EURETINA 2019, and recent public results showed that treatment with 2.0 mg OPT-032 in combination with ranibizumab in patients with NVAMD achieved a statistically significant average visual acuity compared with ranibizumab alone, with a good safety profile indicating that the combination of VEGF-A, VEGF-C and VEGF-D to inhibit CNV as a treatment for NVAMD may have great potential (Jackson et al., 2023). Two Phase III clinical trials (ShORe trial NCT04757610; COAST trial NCT 04757636) are ongoing.

BAT5905 (Bio-Thera) is a recombinant humanized full monoclonal antibody targeting VEGF. A Phase I clinical trial (NCT04151212) to evaluate the safety and pharmacokinetics of BAT5905 (single dose starting from 0.3 mg, intravitreal injection (IVT) in patients with NVAMD when the injection dosage escalates is underway. The secondary objective is to evaluate the immunogenicity profile. To date, no results have been reported.

### 5.2 Novel targets

#### 5.2.1 Platelet-derived growth factor

Platelet-derived growth factor (PDGF) mediates the recruitment and maturation of pericytes, providing VEGF-A and other growth factors via paracrine signaling (Caporarello et al., 2019). Studies have suggested that limitations of anti-VEGF-A therapy may be related to pericytes (Jo et al., 2006) Pegpleranib (Fovista®, Ophthotec) is aPEGylated DNA aptamer that binds to PDGF-BB and blocks PDGF binding to PDGF receptor-β (PDGFRβ) on pericytes, resulting in the death of cells (Jaffe et al., 2016; Jaffe et al., 2017). A Phase IIb clinical trial (NCT01089517) included 499 patients with NVAMD, and the results showed that visual acuity improved by 10.6 letters in the treatment group of pegpleranib 1.5 mg plus ranibizumab 0.5 mg, while an improvement of only 6.5 letters was observed in the ranibizumab monotherapy group (Jaffe et al., 2017). However, the Phase III (NCT01940900) trial failed to replicate the success of the Phase II trial (Rosenfeld and Feuer, 2018). HL-217 is a synthetic inhibitor targeting PDGF-BB: PDGFRβ signaling pathway, which effectively inhibits subretinal neovascularization when applied topically in rodent studies (Kim et al., 2015). A Phase I clinical trial (NCT03648346) has already finished, and HL-217 is now entering Phase II trials (EudraCT number: 2019–000642-35). Rinucumab (an anti-PDGFRβ antibody) coformulated with aflibercept stopped its Phase II clinical trial (NCT02418754) because no visual or anatomical structure improvements were observed compared with aflibercept monotherapy at 3 months (Heier et al., 2020). To date, no strong evidence has shown that anti-PDGF drugs have reinforced benefits in NVAMD treatment.

#### 5.2.2 CCR3

The eosinophil/mast cell chemokine receptor CCR3 is specifically expressed in choroidal neovascular endothelial cells in humans with AMD, and genetic or pharmacological targeting of CCR3 or eotaxin inhibited injury induced CNV in mice (Takeda et al., 2009). A 6-week Phase IIa trial of AKST4290 (Alkahest, Inc.), an orally administered small-molecule CCR3 inhibitor (400 mg twice per day), met the primary endpoint of achieving an increase in BCVA (mean BCVA improved by +7.0 letters; In 24 of the patients, the BCVA was stable or improved, with six (20/7%) gaining 15 letters or more) in patients with NVAMD. AKST4290 was also found to be safe and well tolerated, meeting the secondary endpoint of the trial (Stewart et al., 2022).

#### 5.2.3 Tissue factor

Tissue factor (TF), known as coagulation factor III, is an integral membrane protein that triggers the blood coagulation cascade and is involved in many pathological processes (Harlos et al., 1994); it is also an angiogenic-specific receptor and the target molecule for fVII-targeted therapeutics (Hu et al., 2017). The expression of TF increases in the retinal pigment epithelium in NVAMD patients (Grossniklaus et al., 2002) but not in normal blood vessels (Belting et al., 2004). Anti-TF monoclonal antibodies can predominantly decrease the expression of TF, VEGF, and F4/80 and reduce the area of experimental CNV induced by laser photocoagulation of the retina in mice (Wang et al., 2014). ICON-1 (hl-con1) (Ionic Therapeutics) is an immunoconjugate protein against TF. Basically, it consists of two identical chains of proteins: human Factor VII (fVII) as the targeting domain and human immunoglobulin G1 Fc as the effector domain, functioning as binding to pathological vessels overexpressing tissue factor (Bora et al., 2003; Tezel et al., 2007). Although intravitreous administration of a single 0.3 mg dose of ICON-1 was safe and well tolerated, its biological activity was evident in a Phase I clinical trial (Wells et al., 2018), and the results reported from a Phase II clinical trial (NCT02358889) with improved BCVA as the primary outcome showed that hl-con1 0.3 mg in combination with ranibizumab 0.5 mg did not surpass ranibizumab 0.5 mg monotherapy. Another phase II trial (NCT03452527) showed that the CNV size assessed by OCT angiography in the ICON-1 0.6 mg maintenance therapy group did not exceed that in the ICON-1 0.6 mg combination therapy group (with aflibercept 2 mg). A new tissue factor monoclonal antibody, ICON-4, a potential therapeutic for NVAMD, is now beginning to enter clinical trials.

#### 5.2.4 Fibroblast growth factor 2

Fibroblast growth factor 2 (FGF-2) modulates the function of many cell types by binding heparin and heparan sulfate, stimulating angiogenesis, normal wound healing, and tissue growth (Nugent and Iozzo, 2000). RBM-007 (Ribomic, Inc.) is an anti-FGF-2 aptamer that inhibits angiogenesis and scar formation (Matsuda et al., 2019; Nakamura, 2021). A Phase II trial (TOFU trial, NCT04200248) compared monthly IVT of RBM-007 alone and RBM-007 combined with aflibercept dosed every other month for 16 weeks. However, no results have been reported yet.

#### 5.2.5 Angiopoietin-2

The angiopoietin/tyrosine kinase with immunoglobulin and epidermal growth factor homology domains (Ang/Tie) pathway plays an important role in regulating vascular development and maintenance (Maisonpierre et al., 1997; Saharinen et al., 2008; Thomas and Augustin, 2009). Angiopoietin-1 (Ang-1) binds and phosphorylates the Tie2 receptor to maintain stable vasculature and homeostasis, and angiopoietin-2 (Ang-2) inversely inhibits the Ang-1/Tie2 axis, inducing inflammation and pathologic new vessels (Hackett et al., 2000; Biel and Siemann, 2016). Nesvacumab (REGN910/SAR307746) is a fully human immunoglobulin G1 (IgG1) monoclonal antibody that specifically binds and inactivates Ang-2 with high affinity. A Phase I clinical trial (NCT01688960) of nesvacumab combined with aflibercept showed positive results in vision and anatomical structure improvement. However, in a Phase II clinical trial (the ONYX study, NCT02713204) comparing the efficacy of nesvacumab in combination with aflibercept and aflibercept monotherapy, the results did not suggest a significant difference (Brown et al., 2022). A Phase III clinical trial will not be conducted. However, clinical evidence still suggests a potential benefit of targeting the angiopoietin/Tie pathway and VEGF-A over anti-VEGF-A monotherapy alone (Sharma et al., 2020; Heier et al., 2021), as we will describe below.

### 5.3 Multitargets

Ang-2 and VEGF-A are simultaneously blocked with Faricimab (Hoffmann-LaRoche), a bispecific antibody that binds to both simultaneously and independently (Regula et al., 2019). A Phase II clinical trial (STAIRWAY NCT0303888) administered extended dosing regimens in seventy-six treatment-naïve participants with NVAMD. After the initial dose of faricimab 6 mg was administered by intravitreal injection (IVT) once every 4 weeks (Q4W), two different extended treatment plans of Q16W and Q12W were performed and compared with ranibizumab 0.5 mg IVT Q4W. The results showed that the visual improvement in extended treatment with faricimab was comparable to the effect of monthly ranibizumab use, and the mean total numbers of injections through Week 52 were 6.7, 6.2, and 12.9, respectively, which supports its potential to provide sustained efficacy (Khanani et al., 2020). In Phase III clinical trials TENAYA (NCT03823287) and LUCERNE (NCT03823300), patients were randomly assigned (1:1) to two groups:IVT of faricimab 6.0 mg up to every 16 weeks and aflibercept 2.0 mg every 8 weeks. In 1,329 patients with NVAMD, the improvement of BCVA with group of faricimab was noninferior to group of aflibercept. The mean change of letters in TENAYA was 5.8 letters and 5.1 letters in two group respectively, the treatment difference was 0.7 letters; In LUCERNE, the mean change of letters was 6.6 letters and 6.6 letters respectively, the treatment difference 0.0 letters; rates of ocular adverse events were comparable between groups (Heier et al., 2022). The results were promising to show that faricimab administered at interval up to 16-week has the potential to meaningfully extend the time between injections, thereby reducing the treatment burdens in patients (Chia and Keane, 2022; Heier et al., 2022). Vabysmo ^®^ (faricimab, Genentech) was approved by the FDA in January 2023.

IBI302 (efdamrofusp alfa, Innovent Biologics, Suzou Co. Ltd.), an innovative bispecific decoy receptor fusion protein, inhibits both VEGF and the complement cascade connected by the fc region of human immunoglobulin (Ren et al., 2016; Wang et al., 2018). The Phase I clinical trial (NCT03814291) showed a mean improvement in BCVA of +6.0 letters by Day 29 and 6.1 letters by Day 43 after a single injection of IBI302 (Yang S. et al., 2022). Another Phase I clinical trial (NCT04370379) conducted the safety and tolerability evaluation of a multidose IVT of IBI302 every 4 weeks with a 3+ PRN regimen (initial 3 injections loading doses followed by IVT *pro re nata*). The results were first announced at the Annual Meeting of the American Academy of Ophthalmology (AAO) in January 2020 that IBI302 improved BCVA in patients with NVAMD, decreased central retinal thickness (CRT), and reduced CNV leakage and area, indicating that the overall safety and efficacy characteristics of IBI302 were the same as those of currently available clinical anti-VEGF drugs. A dose escalation Phase II trial (NCT04820452) is ongoing.

RC28-E (RemeGen) is a decoy receptor trap that blocks both VEGF and FGF-2. In preclinical studies, RC28-E was suggested to be more effective in inhibiting pathological angiogenesis than other VEGF antagonists in CNV (Jiang et al., 2018; Jiang et al., 2020). The Phase I and phase II clinical trials (NCT03777254, NCT04270669) were dose escalation studies of RC28-E (0.5–2 mg IVT loading dose plus PRN treatment) for up to 48 weeks. To date, no public results have been reported.

### 5.4 Tyrosine kinase receptor inhibitors (TKIs)

To date, most of the emerging therapies entering clinical trials discussed in this section are administered via intravitreal injection and have been applied as regular, ongoing treatments in monthly “treat-and-extend” (T&E) or *pro re nata* (PRN, or “as needed”) regimens (Mettu et al., 2021). Hence, replacement of IVT injections by noninvasive drug delivery, such as topical or oral agents, is under development (Zeitz and Joussen, 2017). Large peptides less able to penetrate ocular tissue membranes make topical administration challenging. Tyrosine kinase receptor inhibitors are cell-penetrating peptides that enhance cellular and tissue penetration, showing potential as topical anti-VEGF agents (Pescina et al., 2018). **Pazopanib** is a multitargeted TKI that inhibited VEGFR 1, 2, and 3 and PDGF signaling and reduced CNV size in a preclinical animal model (Takahashi et al., 2009). In a Phase II trial (NCT00612456), BCVA in patients with NVAMD was improved by topical administration of 5 mg mL^-1^ and 3 times daily of pazopanib; nevertheless, pazopanib was not able to achieve the primary endpoint in a Phase IIb trial (NCT01134055) at Week 52, neither did it reduce the number of ranibizumab injections by 50% nor was it inferior to ranibizumab (Danis et al., 2014; Csaky et al., 2015). Acrizanib (LHA510, Alcon) is a tyrosine kinase inhibitor administered via suspension eye drops (Adams et al., 2018). A Phase II clinical trial (NCT02355028) evaluated the efficacy of 84 successive days of topically administered LHA510 compared to vehicle in reducing the number of patients requiring ranibizumab IVT therapy for the recurrence of CNV. Initial results showed that the LHA510 group failed to gain superiority over the vehicle group at the endpoint (Poor et al., 2022). A Phase IIa trial (NCT02222207) of another therapeutic agent regorafenib which inhibits VEGF-R 2/3 and PDGFR, as a result of losing 2.4 EDTRS letters at 12 weeks, was discontinued (Joussen et al., 2019). Although the results for these drugs have not been favorable, the VEGFR2 tyrosine kinase inhibitor **PAN-90806** (NCT0347937) with an improved formulation showed safety and biological response in NVAMD as monotherapy, accomplishing the goal of 51% of participants not receiving rescue anti-VEGF injections. These results were presented at the American Academy of Ophthalmology annual meeting.

Vorolanib (X-82, CM082) is an oral multikinase inhibitor that targets VEGFR, PDGFR and CSF1R. A Phase I clinical trial (NCT03038880) of dose escalation showed improvement in BCVA and CRT in recruited subjects (Jackson et al., 2017)). The results from a Phase II clinical trial (NCT02348359) showed that patients treated with X-82 gained noninferiority efficacy in improving visual acuity compared to those who received placebo and had a lower IVT burden. Some patients could even completely stop receiving IVT of anti-VEGF therapy. However, X-82 did not achieve sufficient risk-benefit, and its systemic toxicity limits its clinical application (Cohen et al., 2021).

GB-102 is a novel depot formulation of sunitinib malate, which is a bispecific tyrosine inhibitor of VEGFR-2 and PDGFRβ. The drug is encapsulated in a biodissolving polymer nanoparticle capsule to achieve a sustained release effect at 2 doses per year for the treatment of NVAMD. A Phase I clinical trial (NCT03249740) showed favorable tolerability and safety, with some patients maintaining stable vision up to 8 months. However, vision reduction occurred in the 2 mg therapeutic dose group due to the dispersion of particles into the anterior chamber. A new manufacturing process to eliminate particle dispersion and incomplete aggregation is under development. The purpose of the Phase IIb trial (NCT03953079) was to evaluate the safety and duration of the effect of GB-102 by measuring the time to first rescue treatment with IVT of GB-102 1 mg or 2 mg at Baseline and then 1 mg at month 6, compared to aflibercept administered every 2 months, and the patients recruited was received prior anti-VEGF therapy. The results showed that both doses of GB-102 elevated the visual acuity; interestingly, the primary outcome was 5 months (3–8 months) and 4 months (3–4 months) in group of GB-102 1mg and 2 mg administrated at Baseline respectively.

### 5.5 Gene therapy

AAV2-sFLT01 (Genzyme, Sanofi Company) is an AAV2 vector expresses SFLT01, a VEGF-neutralizing protein containing the VEGF/PlGF binding domain of human VEGFR1/Flt-1 (hVEGFR1) coupled to a Fc portion, and domains chained by polyplycine linker (Bagley et al., 2011). A Phase I clinical trial (NCT01024998) showed that intravitreous injection of AAV2-sFLT01 seemed to be safe and well tolerated at all doses (Heier et al., 2017), and NVAMD patients maintained stable visual improvement. In 19 patients, among 11 patients with reversible intraretinal or subretinal fluid at baseline, six showed a significant reduction of fluid and improvement in vision, whereas five did not. However, although the AAV2-sFLT01 group received a lower retreatment rate of ranibizumab compared with the control group, anti-VEGF drugs were still used as a supplement. Therefore, the significant advantages of a single injection of gene therapy have not been realized.

RGX-314 (Regenxbio Inc.) RGX-314 is being developed as a potential novel single gene therapy treatment for NVAMD via applanation of a novel NAV-AAV8 vector containing a gene for a monoclonal antibody fragment to neutralize VEGF (Khanani et al., 2022). Two phase II Randomized, dose-escalation trials (NCT04514653, NCT04832724) have been designed to evaluate whether RGX-314 gene therapy is effective, safe, and tolerable in subjects with NVAMD, no result has been public reported yet.

**FIGURE 3:**
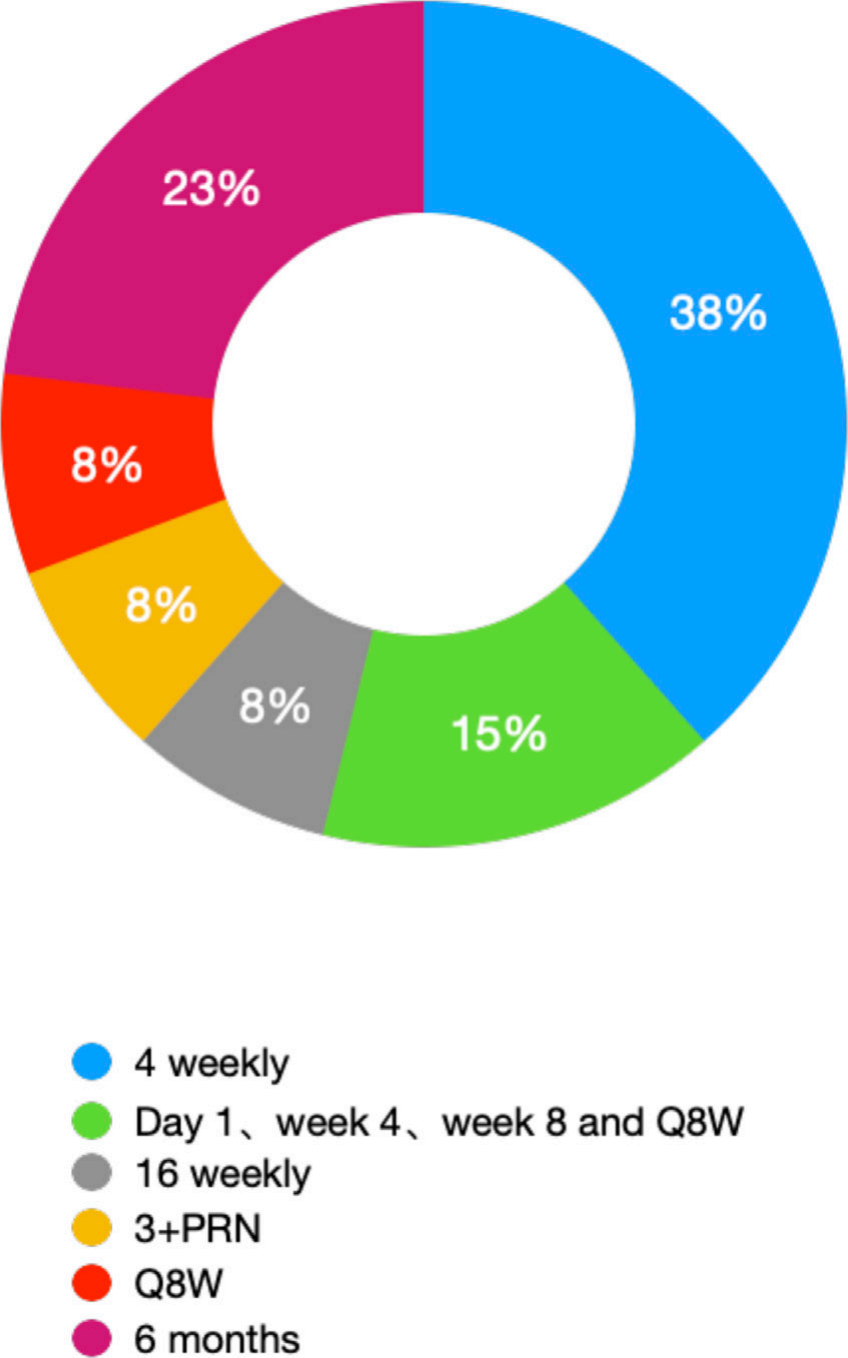
Ratio of different IVT gaps of treatment for NVAMD (statistic result form Table 2).

We selectively summarized and categorized the clinical trials of novel therapies for NVAMD (Table 1). Most of the clinical trials described above were stopped before Phase II/III; some succeeded but only showed noninferiority of efficacy compared with the currently available anti-VEGF monotherapy, which means it is not able to replace its counterpart. To maintain efficacy, patients must receive intraocular injections for life, usually every four to 12 weeks (Table 2 and Figure 3). Due to the treatment burden, patients often experience a decline in vision with reduced frequency of treatment over time. Thus, there are substantial unmet needs in the treatment of NVAMD.

**TABLE 1:** A selection of clinical trials of novel therapies for NVAMD.

Novel antagonist of VEGF-A		Route of administration	Dose regime	Results	Trials	Trial number	Reference
Abicipar pegol (abicipar) (Allergan)	Targeting VEGF-A with lower molecular weight, higher affinity, and longer ocular half-life	IVT	2 mg injected Day 1, Week 4 and Week 12, then every 12 weeks until Week 96	Abicipar showed comparable BCVA and CRT with fewer injections than Ranibizumab up to 52 weeks, but failed to gain FDA approval in 2020	Phase I/II Phase II Phase III	NCT01086761 NCT01397409 NCT02181504 NCT02462486 NCT02462928	Ferro Desideri et al. (2020); Kunimoto et al. (2020)
BAT5906 (Bio-Thera Solutions)	Recombinant anti-VEGF humanised monoclonal antibody	IVT	Single dose escalation starting from 0.3 mg	No results have been reported	Phase I	NCT04151212	N/A
OPT-032	Fusion protein of Fc fragments of VEGFR-3 and human IgG1 for binding VEGF-C and D	IVT	0.5 mg, 2.0 mg OPT-032 combined with ranibizumab IVT 4 weekly compared with sham injection with ranibizumab	At 24 weeks, mean BCVA in 2.0 mg OPT-032 group was significantly superior to sham. Adverse events were similar across groups	Phase I/II2a Phase IIb	NCT003345082	Jackson et al. (2023)
					Phase III	NCT04757610 NCT04757636	

Novel target		Route of administration	Dose regime	Results	Trials	Trial number	Reference
Pegpleranib/Fovista (Ophthotech)	PEGylated DNA aptamer with selective anti-PDGFβ action	IVT	In phase II trial 1.5 mg pegpleranib plus ranibizumab 0.5 mg IVT every 4 weeks	Visual acuity improved by 10.6 letters in the treatment group of pegpleranib 1.5 mg plus ranibizumab 0.5 mg than ranibizumab monotherapy at 24 weeks. Not replicated in Phase III trial	Phase I Phase II Phase III	NCT01089517 NCT01940900	Jaffe et al. (2016); Jaffe et al. (2017)
HL-217	Synthetic inhibitor PDGF-BB	Topical	HL217 applied BID or TID compared with placebo	no results reported	Phase I Phase II	NCT03648346 EudraCT number: 2019-000642-35	N/A
Rinucumab-Aflibercept	an anti-PDGFRβ antibody co-formulated with aflibercept	IVT	Rinucumab 1 mg/3 mg and aflibercept 2 mg IVT every 4 weeks compared with aflibercept 2 mg mono therapy	No visual and anatomical structure improvements between groups	Phase I Phase II	NCT02418754	Heier et al. (2020)
AKST4290 (Alkahest, Inc.)	Antagonist at CCR3 (receptor for eotaxin)	Oral	AKST4290 400 mg twice daily for 6 weeks	Mean BCVA improved by +7.0 letters in 6-week study	Phase IIa	NCT04331730	Stewart et al. (2022)
ICON-1 (hl-con1) (Ionic Therapeutics)	Factor VII-IgGFc chimeric protein targeting tissue factor	IVT	ICON-1 0.3 mg or 0.6 mg monthly injection combined with ranibuzmab 0.5 mg or aflipercept 2.0 mg	Both BCVA and numbers of CNV size-smaller didn’ t exceed the monotherapy group	Phase I Phase II/IIa	NCT02358889 NCT03452527	Wells et al. (2018)
RBM-007 (Robotic, Inc.)	Anti-FGF-2 aptamer inhibits angiogenesis and scar formation	IVT	Four monthly intravitreal injections of RBM-007 alone or in combination with aflipercept, compared with aflipercept alone	no results reported	Phase I/II Phase II	NCT04200248 NCT04640272 NCT04895293	N/A
Nesvacumab(REGN910/SAR307746)	Human IgG1 monoclonal antibody binding and inactivating Ang-2 with high affinity	IVT	REGN910 2 mg or 4 mg every 4 weeks on Day 1, Week 4, and Week 8 for 3 initial doses followed by every Week 8 (Q8) dosing beginning at Week 16 up to Week 32	No significant difference between groups	Phase I	NCT01688960 NCT02713204	Brown et al. (2022), Sharma et al. (2020), Heier et al. (2021)

Multi-targets		Route of administration	Dose regime	Results	Trials	Trial number	Reference
RC28-E (RemeGen)	A chimeric decoy receptor trap fusion protein by dual block of VEGF and FGF-2	IVT	Dose escalation study 0.5–2 mg intravitreal injections up to 48 weeks	No results reported so far	Phase I Phase I/II	NCT03777254 NCT04270669	N/A
IB1302 (efdamrofusp alfa, Innovent Biologics, Suzhou Co. Ltd.)	Novel bispecific decoy receptor fusion protein designed with domains to inhibit both VEGF and the complement cascade and are connected by the fc region of human immunoglobulin	IVT	In Phase I clinical trial, injection of IBI302 every 4 weeks (3 injections) followed by PRN dosing	IBI-302 improved BCVA, CRT, and reduced CNV leakage and area, were non- inferior with now the clinical available anti-VEGF drugs	Phase I Phase II	NCT04370379 NCT04820452	Ren et al. (2016)
Faricimab (RO6867461;RG7716) (Hoffmann-LaRoche)	Simultaneous blockade of Ang-2 and VEGF-A with the bispecific antibody via both simultaneous and independent binding	IVT	In Phase III trial, intravitreal faricimab 6.0 mg up to every week compared with aflibercept 2.0 mg every 8 weeks	BCVA change from baseline with faricimab was non-inferior to aflibercept	Phase II Phase III Faricimab was approved by FDA in January 2023	NCT0303888 NCT03823287 NCT03823300	Khanani et al. (2020), Heier et al. (2022), Chia and Keane (2022)

TKI		Route of administration	Dose regime	Results	Trials	Trial number	Reference
Pazopanib	Multi-targeted TKI which inhibits VEGFR 1, 2, 3 and PDGF signaling	Topical	5 mg mL^-1^ eye drops applied 3 times daily	BCVA in patients with NVAMD was improved, but not non inferior to ranibizumab in a Phase IIb trial	Phase I Phase II/II	NCT00612456 NNCT01134055	Danis et al. (2014), Csaky et al. (2015)
Acrizanib(LHA510) (Alcon)	Small-molecule TKI blocking kinase activity of all 3 VEGFRs	Topical	2% LHA510 compared with topical vehicle twice daily to reduce the need of IVT anti-VEGF therapy	LHA510 failed to gain the superiority than the vehicle group at the end point	Phase I Phase II	NCT02355028	Poor et al. (2022)
PAN-90806	A VEGFR2 tyrosine kinase inhibitor	Topical	One drop of PAN-90806 once daily for 12 weeks	51% participants did not receive rescue anti-VEGF injections	Phase I/II	NCT0347937	N/A
CM082 (also called X-82) (AnewPharma)	Oral multi kinase inhibitor that targets VEGFR, PDGFR and CSF1R	Oral	Oral tablet twice (25–50 mg) daily for 2 weeks followed by 2 weeks off in 4-week cycles. The treatment period is tentatively set at 1 year	X-82 gained non-inferiority efficacy in improvement of visual acuity compared to placebo, also with less IVT burden, but the systemic toxicity limits application	Phase I Phase II	NCT03038880 NCT02348359	Cohen et al. (2021)
GB 102	Bispecific tyrosine inhibitor on VEGFR-2 and PDGFRb	IVT	GB 102 administered every 6 months as compared to aflibercept administered every 2 months in subjects	Two doses of GB102 elevated the visual acuity, however no data of the efficacy compared with aflibercept has been reported	Phase I Phase II	NCT03249740 NCT03953079	N/A

Gene therapy		Route of administration	Dose regime	Results	Trial status and outcome	Trial number	References
AAV2-sFLT01 (Genzyme, Sanofi Company)	AAV2 vector expressing the VEGF neutralising protein SFLT01	IVT	Single injection; Four dose-ranging cohorts (cohort 1, 2 × 108 vector genomes (vg); cohort 2, 2 × 109vg; cohort 3, 6 × 109vg; cohort 4,2 × 1010 vg, n-3 per cohort)	Up to 52 weeks, AAV2-sFlT01 groups obtained a lower pretreatment rate of ranibizumab, anti-VEGF drugs was still a supplement. Some temporary systemic effects	Phase I	NCT01024998	Heier et al. (2017)
RGX-314(Regenxbio Inc.)	NAV-AAV8 vector containing gene for monoclonal antibody fragment to neutralise VEGF	Suprachoroidal space (SCS)	Single injection. Three doses compared with ranibizumab IVT, evaluating change in BCVA at 40 weeks	No results reported	Phase I Phase II	NCT04514653 NCT04832724	N/A

**TABLE 2:** IVT injection gap of part of clinical trials for NVAMD (A deuterogenic paradigm of column Dose regimen of Table 1).

Gap of IVT injection	Selectively drugs		Numbers
4 weekly	OPT-302	5	
	Pegpleranib		
	Rinucumab		
	ICON-1		
	RC28-E		
Day 1, week 4, week 8 and Q8W	Abicipar	2	
	Nesvacumab		
16 weekly	RBM-007	1	
3+ PRN	IB1302	1	
Q8W	Fricimab	1	
6 months	GB102	3	
	AAV2		
	RGX		

## 6 NVAMD drug delivery systems

To realize or enhance the activities of the therapeutics listed above, suitable delivery systems, especially long-term delivery systems for small molecular drugs or antibodies, are required (Campochiaro and Akhlaq, 2021). Various types of biomaterials, including hydrogels and microparticles, have been designed and fabricated as delivery systems for the long-term delivery of neovascularization inhibitors, e.g., anti-VEGF-A and recombinant fusion proteins, as replacements for intravitreal administration (Iyer et al., 2019; Jiang et al., 2020). However, due to the unique microenvironment of NVAMD and the strict requirements of the formulations for intravitreal injection, many of these systems are still being evaluated in preclinical assays, and there is a wide gap to be surmounted; they need to preserve the bioactivities of therapeutics; have a large payload (capacity), targeted delivery, sustained release (long period for drug delivery), and biocompatibility; and remain optically clear across the entire implanted or injected period (Kulkarni and Kuppermann, 2005; Fleckenstein et al., 2021).

**FIGURE 4:**
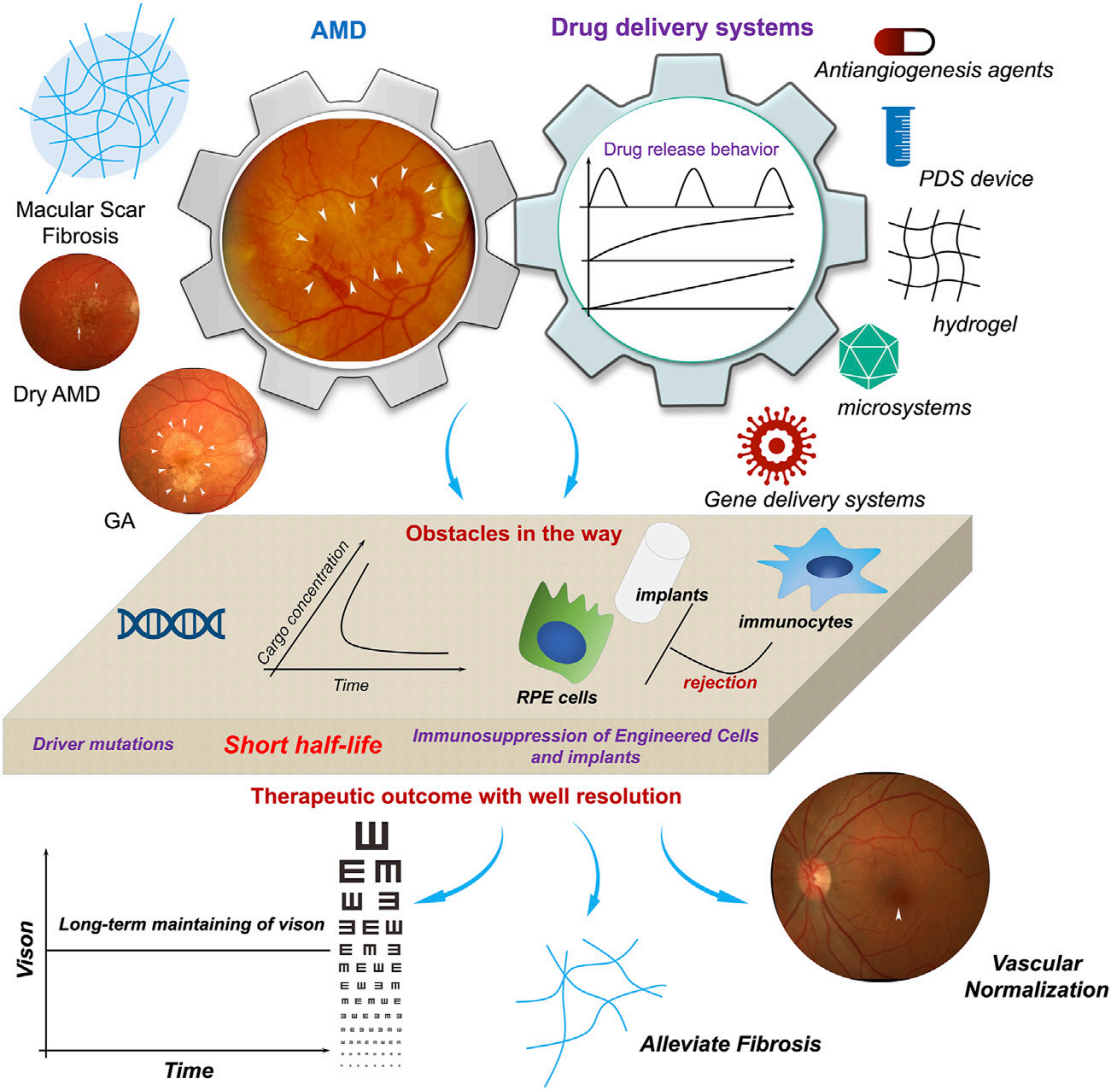
The potentials and obstacles of drug delivery systems utilized in AMD therapy. With finish machining, prolonged activities of therapeutics in AMD therapy as well as gene intervention can be achieved. However, the uncertainty of gene mutation controlling the initiation and progression of AMD, the short half-life of anti-VEGF-based therapeutics, and immunosuppression of host to engineered cells or biomaterials-based implants, etc. are impeding the realization of well management of AMD. It spurs the development of more ideal delivery system for AMD therapy. Adapted with permission from (Nat. Commun. 2019, 10, 3347) (Handa et al., 2019).

The development and performance of port delivery systems (PDSs) in intravitreal delivery of anti-VEGF for long-term control of the progression of NVAMD must be mentioned. The release of the Phase I and Phase II clinical trial results of PDSs has demonstrated that PDSs not only realize long-term delivery of anti-VEGF with a significant reduction in administration time but also spur the further development of long-term delivery systems for intravitreal administration. Although it has been reported and highlighted by some other reviews, the importance and significance of its clinical trials are worthy of brief mention. PDSs are fabricated from nondegradable materials, mainly silicon-based walls and metal-based outlets, to form a hollow sealed tube with 20 µL space for cargo loading. A PDS can be implanted and fixed in the sclera, and the tube is inserted into the vitreous body at a depth of 4 mm; the outside end is an extrascleral flange with a self-sealing septum in the center for further refilling (Campochiaro et al., 2019), and the end inside the vitreous body is plugged with a metal element for controlled release of the cargo, which is typically anti-VEGF-A and ranibizumab. The most predominant advantage of a PDS is its performance in prolonging the interval between intravitreal injections. The median time to first refill is approximately 15.0 months when the PDS is initiated with a 100 mg/mL dosage (20 μL, 2 mg in total), and in general, the standard injection frequency is monthly injections of 0.5 mg ranibizumab. The visual and anatomic outcomes of the PDS 100 mg/mL arm were comparable to those of the monthly injection arm. From the aspect of pharmacokinetics, ranibizumab can still be detected in the serum, and the mean concentration is approximately 50.8 pg/mL 16 months after the implantation of the PDS (100 mg/mL) (Khanani et al., 2021). This further demonstrates the capacity of PDS in the long-term release of antibodies. The results of Phase I and Phase II clinical trials of PDSs further established the criteria for long-term intravitreal delivery of biomacromolecules (including anti-VEGF and recombinant fusion proteins, although the recombinant fusion proteins have not been loaded into PDSs and evaluated for their potential in prolonging alleviation of NVAMD): refillability; median time to first refill, ∼15.0 months; comparable NVAMD control with monthly injection of 0.5 mg ranibizumab; and low probability of adverse events. Under these criteria, some reported systems for biologics (biomacromolecules) delivery still need to be further improved (Figure 4).

### 6.1 Hydrogel

The vitreous body contains more than 95% water and approximately 1% collagen and matrix, which can be considered a hydrogel system. Therefore, the utilization of hydrogels for biologic delivery is considered a suitable choice. Some promising results have been obtained in preclinical assays (Wang and Han, 2017; Ilochonwu et al., 2020). In theory, biologics can be loaded by hydrogels according to the solubility of the biologics. Furthermore, the *in situ* gelation properties impart the hydrogels with injectability, which not only favors intravitreal injection but also favors the loading of biologics with hydrogels by simple mixing. Among these injectable hydrogel systems, thermoresponsive hydrogels are promising. Hydrogels formed by block copolymers, e.g., PLGA-PEG-PLGA, PCL-PEG-PCL, PDLLA-PEG-PDLLA, have been reported as thermoresponsive hydrogels, and the sol-gel transition temperature can be fine-tuned at 37°C, the body temperature (Ballios et al., 2010). In an *ex vivo* environment (room temperature, 25°C), the hydrogels exhibit a solution form, and the biologics can be well dissolved and mixed with the hydrogel solution. After intravitreal injection, under the circumstances of body temperature, the hydrogel undergoes a sol-gel transition to form a hydrogel *in situ*, which encapsulates the biologics inside the network structure of the hydrogel, thus realizing controlled release of biologics (Gao et al., 2023). In ideal cases, these types of hydrogels are the most promising delivery systems for long-term delivery intravitreal injections. However, the drawbacks of the injectable hydrogels are also obvious. One concern is achieving slow release of the cargos; the release rate and duration time are controlled by the degradation of the hydrogels. Most hydrogel systems formed by block polyesters degrade less than 1 month after *in vivo* injection (Lin et al., 2021). Another issue that needs to be considered is whether the molecules of the degraded hydrogels aggravate the inflammation of the macular microenvironment. Both the PLGA- and PDLLA-based block copolymers degrade into lactic acid, which can exacerbate the inflamed microenvironment during the progression of NVAMD. Although an aflibercept-loaded thermoresponsive hydrogel has demonstrated a 6-month persistent release of aflibercept, from the perspective of long-term delivery of biologics, hydrogel systems still have to be further optimized.

### 6.2 Microparticles

Beyond macrobiologics, small molecular inhibitors are also promising candidates for inhibiting the progression of neovasculature, e.g., tyrosine kinase inhibitors (TKIs) (Tsujinaka et al., 2020). Many types of TKIs have been developed, including single-target and multitarget. Although most TKIs are fabricated for oral administration, the blood-eye barrier or blood‒retinal barrier and the inherent hydrophobic characteristics of TKI molecules dramatically reduce the bioavailability of TKIs in the area of focus of NVAMD. Meanwhile, it has been found that the degradation of some biomaterials approved by the FDA for implantation can be sustained for more than 2 years (PCL and PDLLA). The hydrophobic nature of these biomaterials favors the encapsulation of TKIs for long-term delivery. Sunitinib-loaded poly (ethylene glycol) methyl ether-b-poly (lactide-co-glycolide) (mPEG-PLGA) microparticles have achieved 6 months of sustained release of sunitinib with strong inhibition of type II CNV as well as alleviation of inflammation (Tsujinaka et al., 2020). In addition, fabricating small molecular drugs into microcrystals and combining them with hydrogels also achieved sustained release of axitinib (ClinicalTrial.gov, ID: NCT03630315), which has also been evaluated in patients with NVAMD. Therefore, microsystems (including microparticles and hydrogels) are more suitable for the long-term delivery of TKIs.

### 6.3 Fillable microparticles for pulsatile drug delivery

To achieve desirable drug delivery, the interactions between cargos and carriers need to be considered (Nazila et al., 2016; Mitchell et al., 2021), particularly the microstructures of the carriers, material compatibility, drug distribution inside the microstructures, and the effect of the technique used to fabricate microsystems on the activities of cargos, especially biologics. If the cargos can be encapsulated into a fully enclosed microbox, not only can the bioactivities of cargos be maintained at their initiating station, but the release behavior of the microbox can also be controlled by the properties of the microbox wall. Recently, a microfabrication method termed SEAL (stamped assembly of polymer layers) has been developed to fabricate this type of microbox (sealed particles) with dimensions of 400 × 400 × 300 μm and a cavity dimension of 200 × 200 × 100 μm (McHugh et al., 2017). The most interesting feature of this microbox system is the realization of pulsatile drug delivery after mixing microboxes with different degradation behaviors. The pulsatile drug delivery performance was verified by encapsulating OVA and STING agonists to inhibit tumor growth *in vivo*. By fine-tuning the molecular weight of the polymer used to construct the wall of the microboxes, a release as long as 100 days can be triggered (Lu et al., 2020). Pulsatile drug delivery behavior is the ideal method for long-term delivery, which can avoid burst release. However, for intravitreal long-term delivery (16 months), the time still needs to be further prolonged.

### 6.4 Matrix scaffold simulation for cell delivery

As AMD develops, it progresses from neovascular AMD to exudative or “wet” AMD and then to atrophic “dry” AMD. During AMD progression, impairment and irreversible cell loss are more or less constant. The primary cell type is retinal pigment epithelium (RPE) cells. RPE cells play a critical role in maintaining vison. In the case of NVAMD, RPE cells, which form a tight, smooth cell layer in normal eyes (Bruch’s membrane), may be ruptured due to expansion of macular edema but are not yet lost. In the late stage of AMD, severe amounts of RPE cells are lost. Thus, cell transplantation or cell therapy has become the only efficient way to rescue vision from blindness (Schnichels et al., 2021; Sugita et al., 2021). In 2015, in the first clinical trial related to use autologous induced-pluripotent derived stem cell (iPSC) transplants as treating NVAMD, there was only one subject (Garber, 2015). Even with favorable preclinical experiments and an intact transplanted iPSC derived RPE cell sheet under the retina 1 year after surgery, no alterations of BCVA were observed (Kamao et al., 2014; Mandai et al., 2017a; Mandai et al., 2017b). To realize cell transplantation, a scaffold for cell delivery is required to avoid draining the implanted cells. Human-vitronectin-coated polyester membranes have been evaluated for their performance as RPE cell scaffolds. RPE cells can be derived from human embryonic stem cells (hESCs). The cell density of the implant patch was ∼6,000 cells per mm^2^. The cells cultured in the patch can be induced to differentiate and form a barrier that is as tight as the native configuration. The polyester used in this patch is 10-μm thick polyethylene terephthalate (PET), with a 0.4-μm pore size at a density of 1 × 10^8^ pores/cm^2^ (Sterlitech, Kent, Washington, USA) (da Cruz et al., 2018). Vitronectin is a multifunctional glycoprotein that can connect glycosaminoglycans, collagens, and profibrinolysins in the blood and the extracellular matrix. Thus, the combination of polyester and vitronectin forms a matrix to support the *in vivo in situ* transplantation of RPE cells and their survival for 12 months with only local immunosuppression (Sharma et al., 2019). This strategy can achieve long-term resolution for the control of AMD progression; however, its potential has only been verified in late AMD, in the form of “dry” AMD or geographic atrophy, but not in wet AMD or neovascular AMD. In addition, the immunosuppression possibility of hESC-derived RPE cells, which may result in ocular morbidity, and the effect of the foreign body reaction (FBR) of the scaffold on long-term transplantation also need to be further investigated (Yang Q. et al., 2022).

### 6.5 Gene mutation during AMD

The formation and progression of AMD is considered to be regulated by the combinational interactions of multiple factors: aging is the predominant factor, and it is also the main driver that stimulates the overexpression of VEGF-A in the macular microenvironment in elderly patients (aging induces choriocapillaris depletion and results in the hypoxia of RPE cells, thus promoting the overexpression of HIF-α and VEGF-A by RPE cells and the degeneration of RPE cells and the Bruch’s membrane) (Botto et al., 2021; de Guimaraes et al., 2021), which makes VEGF-A*/HIF-α* -related genes (vegf, vegfr, pdgf, pegf) primary targets for gene therapy (de Guimaraes et al., 2021). AAVs and nonviral delivery systems have been used for the delivery of RNAs or DNAs targeting VEGF-A or hypoxia-inducing factor 1α (HIF-α, a gene controlling the metabolism of cells in the macular microenvironment and promoting the formation of inflammation after transcription), such as lentiviral codelivery of *Streptococcus* pyogenes Cas9 mRNA and RNA targeting VEGF-A or intravitreal delivery of LbCpf1 by AAVs cotargeting VEGF-A and HIF-1α (Koo et al., 2018; Shen et al., 2020; Ling et al., 2021). With the introduction of CRISPR, the therapeutic activities of gene therapy have been further improved. However, beyond VEGF-A, whether the formation and progression of AMD are genetically controlled and which genes are involved, e.g., the K-RAS gene, in promoting the formation of tumors remain to be further clarified. It is speculated that the probability of late AMD containing an inherited feature is ∼70%, while the other ∼30% of uncertainty is ascribed to other factors, including smoking and environmental risk factors (Seddon et al., 2005; Fritsche et al., 2016). With the development of single-cell sequencing and genome-wide association studies (GWASs), the mutations and factors promoting the progression of AMD have been gradually uncovered. Through GWASs, CFH (rs1061170, p.Y402H) 44–47 on chromosome 1 and two neighboring genes, *ARMS2* and *HTRA1*, on chromosome 10, have been identified as two major loci that are closely associated with late AMD (Edwards et al., 2005; Hageman et al., 2005). The CFH variant is mainly associated with drusen area, while the ARMS2-HTRA1 variant is associated with subretinal or sub-RPE hemorrhages (van Asten et al., 2018). Other genes, such as *MMP9, CETP* and *TIMP3*, are mainly linked to NVAMD due to their regulatory capacities in ECM remodeling (Fritsche et al., 2016). The FGD6, HTRA1 and CFH genes play critical roles in regulating oxidative stress and inflammation, which control the progression of angiogenesis, thus promoting the aggravation of NVAMD (Huang et al., 2016). However, to our knowledge, no study has reported the development of therapeutics targeting these uncovered genes. The main targets for gene therapy in AMD are still VEGF-related loci, including VEGFR, PDGF and PEGF, as well as HIF-α. The genetic drivers controlling the initiation and progression of AMD still need to be further investigated. Before the discovery of mature targets for gene intervention in AMD, efforts should focus on the development and optimization of gene delivery systems, e.g., optimization of ionizable lipid nanoparticles (Han et al., 2021) and the development of zwitterionic networks (Blackman et al., 2019; Li et al., 2020). This further promotes the invention of gene therapy, which is also required for suitable gene delivery systems.

## 7 Perspectives

Age-related macular degeneration is a lethal threat to the vision of older people. The progression of AMD has become a major cause of blindness. Although in-depth study of the driver mutations that initiate the formation and promote the progression of AMD is ongoing, the predominant strategies for NVAMD management are VEGF-A-based antiangiogenics. The developed therapeutics are VEGF antibodies or recombinant fusion proteins (aflibercept and conbercept). While TKIs have proven their potential in inhibiting angiogenesis, their useability in intravitreal administration still needs further thorough investigation. Antiangiogenesis via anti-VEGF-A has shown promising clinical outcomes; however, monthly repetitive intravitreal injection of antibodies only achieves limited alleviation of NVAMD, and it is far from achieving essential control of NVAMD progression. Drug delivery systems have been introduced in NVAMD therapy to impart biological therapeutics with long-term delivery capacity. Due to their reported potential in redistribution, drug release control, and off-target release reduction of cargos, biomaterials are expected to be ideal carriers for long-term delivery. However, in NVAMD therapy, only PDSs have achieved long-term performance in delivering VEGF-A antibodies in Phase II trials, while a Phase III clinical trial has been initiated. A PDS is formed by totally undegradable materials (silicon- and metal-based materials) with refillable properties and provides a median time to first refill of ∼15 months. In the case of biomaterial-based delivery systems, injectable hydrogels can only be maintained for one or 2 months. Even hydrophobic polyester (PCL or PLA) can maintain the bulk structure for even 2 years, its inherent hydrophobicity restricts its application to the long-term delivery of hydrophobic small molecular inhibitors, e.g., TKIs. Recently, microboxes fabricated by fine-tuning techniques have been shown to achieve long-term delivery of versatile types of therapeutics, and their potential in intravitreal administration needs further evaluation. Under such circumstances, gene therapy may provide a way to reduce repetitive administration in NVAMD, but the multifactorial character of NVAMD means that gene therapy will require considerable effort to realize desirable therapeutic outcomes.

Besides, numerous studies have shown that both inflammation and oxidative stress-induced retinal pigment epithelial cell damage are involved in the pathogenesis of abnormal vascular development in AMD (AMD). Conventional anti-VEGF therapy (ranibizumab) inhibits the growth of neovascular; however, the adverse effects would occur during the repeated monthly intravitreal injections. Some nano-biomaterials with satisfied biocompatibility and biostability are designed to control the intracellular inflammatory signals by scavenging the reactive oxygen species (ROS), prevent excessive inflammatory activation of innate and adaptive immune cells, and maintain redox balance in biological systems (Yang et al., 2023). In addition, these nanoplatforms can deliver anti-inflammatory agents to the disease region. The anti-VEGF-A antibody-loaded betasone phosphate-based hydrogel (BetP-Gel) not only provides long-term sustained release of anti-VEGF to inhibit retinal vascular proliferation and attenuate choroidal neovascularization, but also scavenge ROS to reduce local inflammation. This BetP-Gel can significantly prolong the effective time of conventional anti-VEGF therapy with a potential clinical application (Gao et al., 2023).

In summary, all the existing challenges inspire us to rethink the innovative development of NVAMD therapeutic strategies, not only to further identify and classify the risk factors that control the initiation and progression of NVAMD (including genetic risk loci and environmental risk factors) but also to design and construct delivery systems with a brand-new perspective from the combination of pathology and materials as well as engineering.

## Abbreviations

A2E, N-retinylidene-N-retinylethanolamine; AMD, age-related macular degeneration; Ang/Tie, angiopoietin/tyrosine kinase; BCVA, best corrected visual acuity; BlinD, basal linear deposits; BrM, Bruch’s membrane; CNV, choroidal neovascularization; CRT, central retinal thickness; Fab, antigen-binding fragment; FGF-2, fibroblast growth factor 2; GA, geographic atrophy; hESCs, human embryonic stem cells; ICAM, intercellular adhesion factor; MAC, membrane attack complex; MNV, macular neovascularization; NVAMD, neovascular AMD; OCT, optical coherence tomography; PDS, port delivery system; PDGF, platelet-derived growth factor; PDGFRβ, PDGF receptor-β; PED, pigment epithelium detachment; PRN, *pro re nata*; RPE, retinal pigment epithelium; SEAL, stamped assembly of polymer layers; T&E, treat-and-extend; TKI, tyrosine kinase inhibitor; VEGF, vascular endothelial growth factor.
